# Supplementary material for: Stories told by corals, algae, and sea-urchins in a Mesoamerican coral reef: degradation trumps succession
Source: PeerJ. 2023 Jan 16;11:e14680. doi: 10.7717/peerj.14680 (PMC9851048; doi:10.7717/peerj.14680)
Supplement: Supplemental Information 1 — RP algae, recruitment promoting algae. Interactive models include main effects and interaction effects [file peerj-11-14680-s001.docx]

**Table S1**. Model structures used for presence of coral recruits in binomial and coral recruit cover proportion in beta regressions. RP algae, recruitment promoting algae. Interactive models include main effects and interaction effects

| Model | Structure |
| --- | --- |
| M1 | Intercept (Null) |
| M2 | Condition |
| M3 | Cover of adult colonies |
| M4 | Cover of CCA |
| M5 | Condition + cover of adult colonies |
| M6 | Cover of CCA + cover of adult colonies |
| M7 | Condition + cover of CCA |
| M8 | Condition × cover of adult colonies |
| M9 | Cover of CCA × cover of adult colonies |
| M10 | Condition × cover of CCA |
| M11 | Condition + cover of adult colonies + cover of CCA |
| M12 | Condition × cover of adult colonies × cover of CCA |
