# Supplementary material for: Stories told by corals, algae, and sea-urchins in a Mesoamerican coral reef: degradation trumps succession
Source: PeerJ. 2023 Jan 16;11:e14680. doi: 10.7717/peerj.14680 (PMC9851048; doi:10.7717/peerj.14680)
Supplement: Supplemental Information 2 — Note that neither null nor univariate (time or Condition) models were considered because the PERMANOVA test demonstrated that both variables significantly affected the response variables (see main text). [file peerj-11-14680-s002.docx]

**Table S2**. Model structures used for framework-building (FB) and non-framework-building (NFB) corals, recruitment-promoting (RP) and non-recruitment-promoting (NRP) algae in the mixed beta regressions. Note that neither null nor univariate (Time or Condition) models were considered because the PERMANOVA test demonstrated that both variables significantly affected the response variables (see main text).

| Model | Structure |
| --- | --- |
| M1 | Time + Condition |
| M2 | Time + Condition + Time × Condition |
| M3 | Time + Condition + Sea urchins |
| M4 | Time + Condition + Sea urchins + Time × Condition |
| M5 | Time + Condition + Sea urchins + Condition × Sea urchins |
| M6 | Time + Condition + Sea urchins + Condition × Sea urchins + Time × Sea urchins |
| M7 | Time + Condition + Sea urchins + Time × Condition × Sea urchins |
| M8 | Time × Condition × Sea urchins |
