# Supplementary material for: Stories told by corals, algae, and sea-urchins in a Mesoamerican coral reef: degradation trumps succession
Source: PeerJ. 2023 Jan 16;11:e14680. doi: 10.7717/peerj.14680 (PMC9851048; doi:10.7717/peerj.14680)
Supplement: Supplemental Information 3 — Boldface indicates the best model for each case. Δ BIC, difference in BIC; w, BIC weight. [file peerj-11-14680-s003.docx]

**Table S3**. Model selection among 12 binomial models for framework-building (FB) and non-framework-building (NFB) corals. Boldface indicates the best model for each case. ΔBIC, difference in BIC; *w*, BIC weight.

| Model | Binomial Regressions | | | | |
| --- | --- | --- | --- | --- | --- |
|  | FB corals | |  | NFB corals | |
|  | ΔBIC | *w* |  | ΔBIC | *w* |
| M1 | 0.00 | **0.590** |  | 0.00 | **0.603** |
| M2 | 4.49 | 0.063 |  | 5.06 | 0.048 |
| M3 | 5.01 | 0.048 |  | 2.09 | 0.212 |
| M4 | 2.68 | 0.155 |  | 4.38 | 0.068 |
| M5 | 9.55 | 0.005 |  | 7.09 | 0.017 |
| M6 | 7.74 | 0.012 |  | 6.57 | 0.023 |
| M7 | 6.91 | 0.019 |  | 9.43 | 0.005 |
| M8 | – | – |  | 10.80 | 0.003 |
| M9 | 12.56 | 0.001 |  | 6.97 | 0.018 |
| M10 | 3.44 | 0.106 |  | 13.42 | 0.001 |
| M11 | 11.87 | 0.002 |  | 11.59 | 0.002 |
| M12 | – | – |  | 24.65 | 0.000 |
