# Supplementary material for: Stories told by corals, algae, and sea-urchins in a Mesoamerican coral reef: degradation trumps succession
Source: PeerJ. 2023 Jan 16;11:e14680. doi: 10.7717/peerj.14680 (PMC9851048; doi:10.7717/peerj.14680)
Supplement: Supplemental Information 4 — Boldface indicates the best model for each response variable. FB, framework-building; RP, recruitment-promoting; Δ BIC, difference in BIC; w, BIC weight. [file peerj-11-14680-s004.docx]

**Table S4**. Model selection among eight models for four response variables. Boldface indicates the best model for each response variable. FB, framework-building; RP, recruitment-promoting; ΔBIC, difference in BIC; *w*, BIC weight.

| Model | Response variables | | | | | | | | | | |
| --- | --- | --- | --- | --- | --- | --- | --- | --- | --- | --- | --- |
|  | Coral | |  | FB corals | |  | Algae | |  | CCA | |
|  | ΔBIC | *w* |  | ΔBIC | *w* |  | ΔBIC | *w* |  | ΔBIC | *w* |
| M1 | **0.00** | **0.891** |  | **0.00** | **0.720** |  | **0.00** | **0.834** |  | 5.30 | 0.062 |
| M2 | 4.98 | 0.074 |  | 2.99 | 0.162 |  | 4.11 | 0.107 |  | **0.00** | **0.881** |
| M3 | 7.34 | 0.023 |  | 4.37 | 0.081 |  | 6.56 | 0.031 |  | 11.28 | 0.003 |
| M4 | 12.21 | 0.002 |  | 7.98 | 0.013 |  | 10.87 | 0.004 |  | 5.83 | 0.048 |
| M5 | 18.31 | 0.000 |  | 15.43 | 0.000 |  | 16.94 | 0.000 |  | 22.07 | 0.000 |
| M6 | 18.78 | 0.000 |  | 16.65 | 0.000 |  | 16.61 | 0.000 |  | 19.93 | 0.000 |
| M7 | 8.95 | 0.010 |  | 6.87 | 0.023 |  | 7.13 | 0.024 |  | 9.82 | 0.007 |
| M8 | 35.44 | 0.000 |  | 30.60 | 0.000 |  | 33.16 | 0.000 |  | 28.57 | 0.000 |
