# Supplementary material for: Stories told by corals, algae, and sea-urchins in a Mesoamerican coral reef: degradation trumps succession
Source: PeerJ. 2023 Jan 16;11:e14680. doi: 10.7717/peerj.14680 (PMC9851048; doi:10.7717/peerj.14680)
Supplement: Supplemental Information 5 [file peerj-11-14680-s005.docx]

**Table S5**. Estimated parameters (± SD) for the eight proposed models and the full mode averaging (indicated in bold typeface), for four response variables (indicated in italics).

| Model | Intercept | Time | Non impacted | Sea urchins | Time × Non impacted | Time × Sea urchins | Non impacted × Sea urchins | Time × Impacted × Sea urchin | Time × Non impacted × Sea urchins |
| --- | --- | --- | --- | --- | --- | --- | --- | --- | --- |
| *Coral cover* | | | | | | | | | |
| M1 | **-1.7044 (0.21707)** | **-0.1058 (0.04939)** | **0.6941 (0.26424)** | **0 (0)** | **0 (0)** | **0 (0)** | **0 (0)** | **0 (0)** | **0 (0)** |
|  |  |  |  |  |  |  |  |  |  |
| *Framework-building coral cover* | | | | | | | | | |
| M1 | -2.3794 (0.34954) | -0.0346 (0.06981) | 0.7576 (0.42405) | 0 (0) | 0 (0) | 0 (0) | 0 (0) | 0 (0) | 0 (0) |
| M2 | -2.6438 (0.39650) | 0.0731 (0.10160) | 1.2510 (0.54340) | 0 (0) | -0.2019 (0.13932) | 0 (0) | 0 (0) | 0 (0) | 0 (0) |
| M3 | -2.3703 (0.35210) | -0.0351 (0.06994) | 0.7317 (0.42814) | 0.0904 (0.10879) | 0 (0) | 0 (0) | 0 (0) | 0 (0) | 0 (0) |
| M4 | -2.6142 (0.40190) | 0.0668 (0.10310) | 1.1912 (0.55380) | 0.075 (0.10980) | -0.1905 (0.14165) | 0 (0) | 0 (0) | 0 (0) | 0 (0) |
| M5 | -2.3684 (0.35538) | -0.0352 (0.07061) | 0.7301 (0.42839) | 0.0817 (0.17050) | 0 (0) | 0 (0) | 0.0091 (0.22566) | 0 (0) | 0 (0) |
| M6 | -2.3419 (0.35837) | -0.0409 (0.07166) | 0.7204 (0.42750) | 0.2221 (0.32805) | 0 (0) | -0.0479 (0.09466) | -0.0047 (0.23203) | 0 (0) | 0 (0) |
| M7 | -2.3441 (0.35472) | -0.041 (0.07113) | 0.7223 (0.42651) | 0.222 (0.28918) | 0 (0) | 0 (0) | 0 (0) | -0.0500 (0.10824) | -0.0483 (0.09637) |
| M8 | -2.7487 (0.49620) | 0.1117 (0.14270) | 1.3212 (0.62180) | -0.3142 (0.83320) | -0.2324 (0.17308) | 0.1278 (0.27547) | 0.4824 (0.89710) | 0 (0) | -0.1626 (0.29506) |
| **Mean (β)** | **-2.4956 (0.39562)** | **0.0144 (0.10139)** | **0.9722 (0.54283)** | **0.0306 (0.09093)** | **-0.0921 (0.11992)** | **-0.00006 (0.014134)** | **0.0010 (0.05781)** | **-0.0003 (0.00889)** | **-0.0005 (0.01538)** |
|  |  |  |  |  |  |  |  |  |  |
| *Algal cover* | | | | | | | | | |
| M1 | 0.7211 (0.1817) | 0.1309 (0.04454) | -0.2443 (0.0936) | 0 (0) | 0 (0) | 0 (0) | 0 (0) | 0 (0) | 0 (0) |
| M2 | 0.8234 (0.20912) | 0.0862 (0.06263) | -0.4541 (0.30021) | 0 (0) | 0.089 (0.08853) | 0 (0) | 0 (0) | 0 (0) | 0 (0) |
| M3 | 0.7118 (0.18144) | 0.1305 (0.04480) | -0.2269 (0.21436) | -0.0457 (0.06843) | 0 (0) | 0 (0) | 0 (0) | 0 (0) | 0 (0) |
| M4 | 0.8101 (0.21137) | 0.0888 (0.06349) | -0.4276 (0.30622) | -0.0382 (0.06932) | 0.0834 (0.09023) | 0 (0) | 0 (0) | 0 (0) | 0 (0) |
| M5 | 0.7000 (0.184) | 0.1330 (0.04517) | -0.2245 (0.21536) | -0.0768 (0.10244) | 0 (0) | 0 (0) | 0.0595 (0.13836) | 0 (0) | 0 (0) |
| M6 | 0.6781 (0.18716) | 0.1385 (0.04586) | -0.2166 (0.21756) | -0.2155 (0.20068) | 0 (0) | 0.048 (0.0595) | 0.0702 (0.14079) | 0 (0) | 0 (0) |
| M7 | 0.6922 (0.18467) | 0.1350 (0.04521) | -0.2200 (0.21744) | -0.1627 (0.17903) | 0 (0) | 0 (0) | 0 (0) | 0.0341 (0.06717) | 0.0506 (0.06103) |
| M8 | 0.7456 (0.26841) | 0.1097 (0.08432) | -0.3622 (0.34795) | -0.2302 (0.48252) | 0.06074 (0.10636) | 0.0545 (0.16038) | 0.1320 (0.52504) | 0 (0) | -0.022 (0.17462) |
| **Mean (β)** | **0.7477 (0.19553)** | **0.1186 (0.05413)** | **-0.3000 (0.21013)** | **0.0082 (0.04292)** | **0.0246 (0.05765)** | **-0.00036 (0.00684)** | **-0.0018 (0.02675)** | **0.00019 (0.00566)** | **-0.0003 (0.00635)** |
|  |  |  |  |  |  |  |  |  |  |
| *CCA cover* | | | | | | | | | |
| M1 | -1.6003 (0.18607) | -0.2264 (0.04902) | 0.1703 (0.20625) | 0 (0) | 0 (0) | 0 (0) | 0 (0) | 0 (0) | 0 (0) |
| M2 | -1.2971 (0.20826) | -0.3693 (0.06952) | -0.4451 (0.29802) | 0 (0) | 0.2703 (0.09473) | 0 (0) | 0 (0) | 0 (0) | 0 (0) |
| M3 | -1.5976 (0.18723) | -0.2266 (0.04932) | 0.1642 (0.20789) | 0.0128 (0.07586) | 0 (0) | 0 (0) | 0 (0) | 0 (0) | 0 (0) |
| M4 | -1.2793 (0.21023) | -0.3742 (0.07026) | -0.4763 (0.30259) | 0.0370 (0.0741) | 0.2780 (0.09594) | 0 (0) | 0 (0) | 0 (0) | 0 (0) |
| M5 | -1.6002 (0.19063) | -0.2268 (0.04963) | 0.1676 (0.20890) | 0.0026 (0.11764) | 0 (0) | 0 (0) | 0.0078 (0.15680) | 0 (0) | 0 (0) |
| M6 | -1.6470 (0.18958) | -0.2173 (0.04985) | 0.1805 (0.20778) | -0.3196 (0.21771) | 0 (0) | 0.1122 (0.06461) | 0.0302 (0.15051) | 0 (0) | 0 (0) |
| M7 | -1.6422 (0.18788) | -0.2172 (0.18788) | 0.1802 (0.20681) | -0.3081 (0.19658) | 0 (0) | 0 (0) | 0 (0) | 0.1212 (0.07353) | 0.1079 (0.06645) |
| M8 | -1.4407 (0.30244) | -0.3189 (0.10293) | -0.2963 (0.37318) | -0.4000 (0.58256) | 0.2152 (0.12259) | 0.1483 (0.19480) | 0.3056 (0.62283) | 0 (0) | -0.1044 (0.20755) |
| **Mean (β)** | **-1.2994 (0.21484)** | **-0.3672 (0.07351)** | **-0.4398 (0.31348)** | **0.0090 (0.07149)** | **0.2661 (0.09453)** | **0.0009 (0.01809)** | **-0.0016 (0.05028)** | **0.0002 (0.00582)** | **0.0004 (0.01735)** |
