# Supplementary figures and images for: Stories told by corals, algae, and sea-urchins in a Mesoamerican coral reef: degradation trumps succession
Source: PeerJ. 2023 Jan 16;11:e14680. doi: 10.7717/peerj.14680 (PMC9851048; doi:10.7717/peerj.14680)

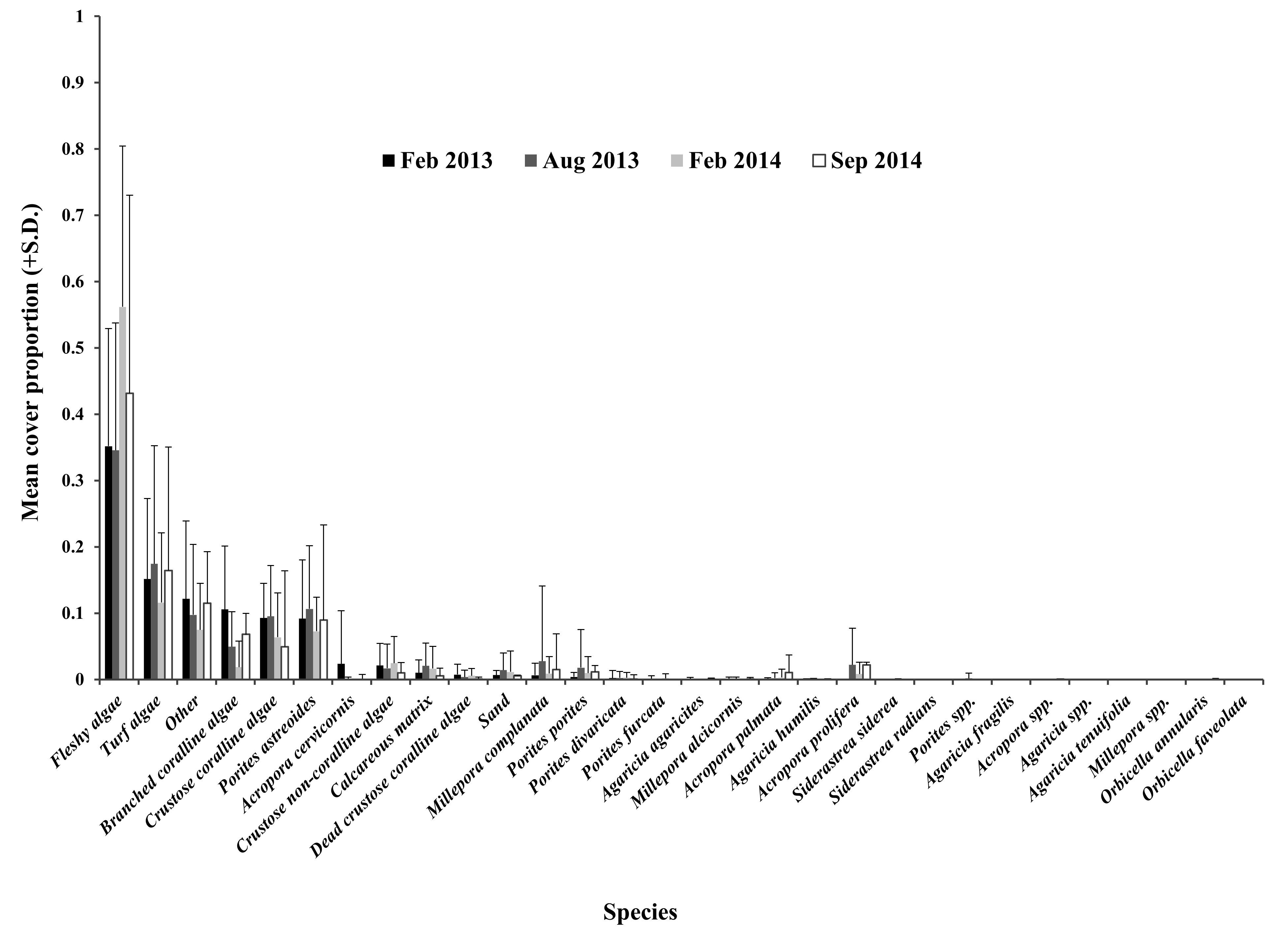

Supplement: Supplemental Information 6 — High SD, with respect to the mean of each group, denotes that the group was absent in most of the quadrats evaluated. [file peerj-11-14680-s006.png]

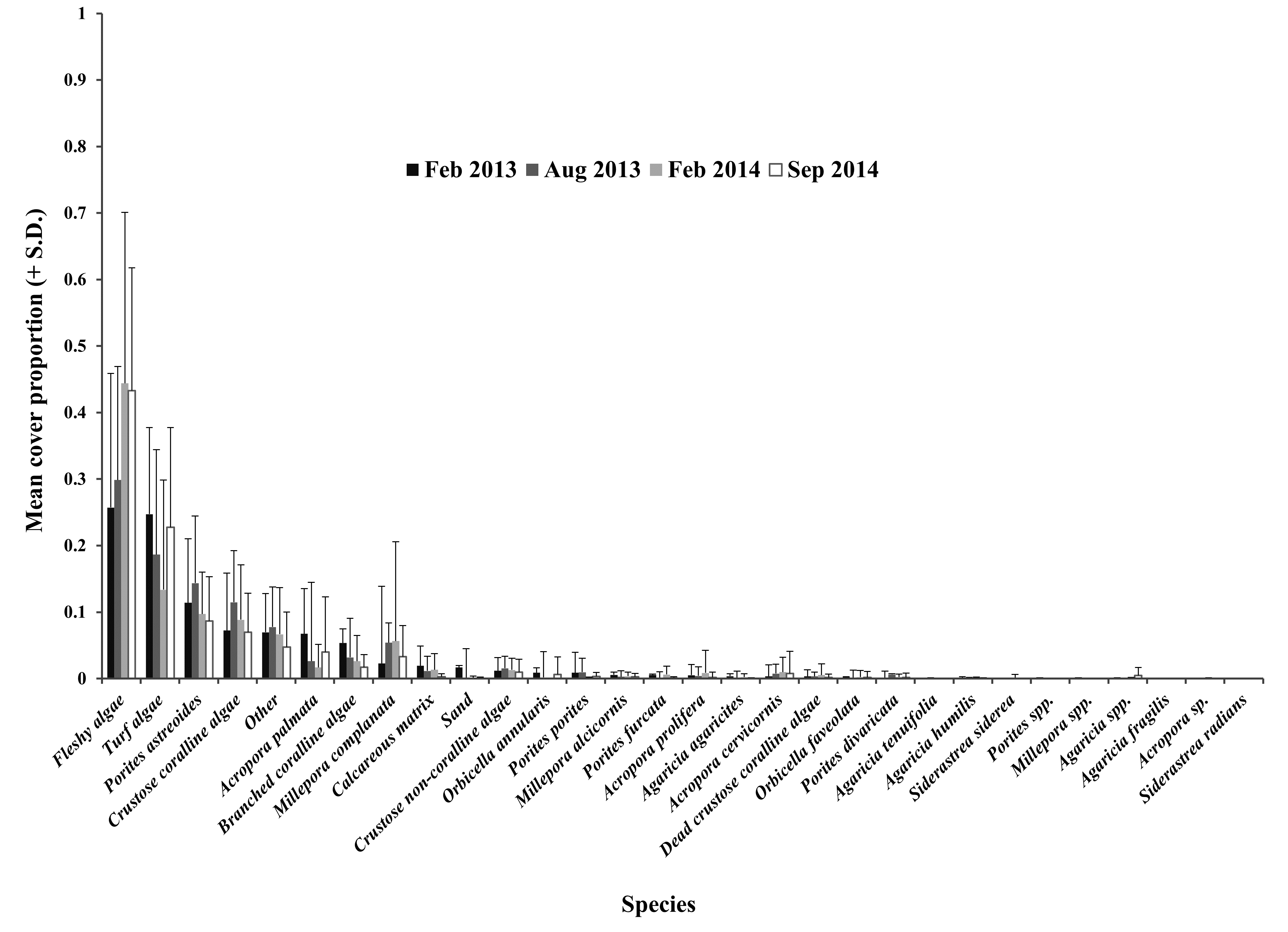

Supplement: Supplemental Information 7 — High SD, with respect to the mean of each group, denotes that the group was absent in most of the quadrats evaluated. [file peerj-11-14680-s007.png]

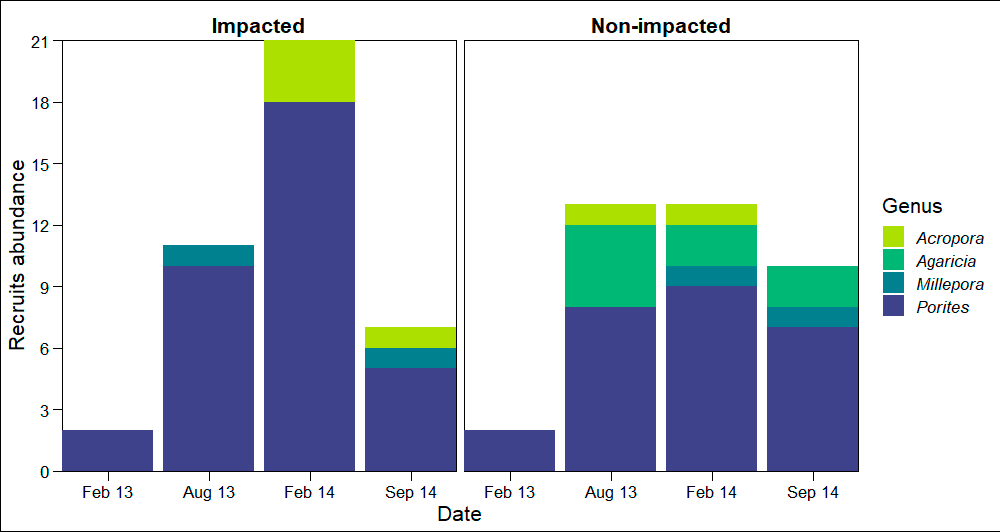

Supplement: Supplemental Information 8 [file peerj-11-14680-s008.png]
